# Cyberbullying and Sleep Disturbances in Adolescents: An Updated Evidence Review

**DOI:** 10.1016/j.acap.2025.103149

**Published:** 2025-09-23

**Authors:** Jason M. Nagata, Elizabeth J. Li, Oliver H. Huang, Colbey Ricklefs, Kyle T. Ganson, Alexander Testa, Aaron Scheffler, Leo Sugrue, Fiona C. Baker

**Affiliations:** Department of Pediatrics (JM Nagata, EJ Li, OH Huang, C Ricklefs), University of California, San Francisco, CA; Factor-Inwentash Faculty of Social Work (KT Ganson), University of Toronto, Toronto, Ontario, Canada; Department of Management (A Testa), Policy and Community Health, University of Texas Health Science Center at Houston, Houston, TX; Department of Epidemiology and Biostatistics (A Scheffler), University of California, San Francisco, CA; Department of Radiology and Biomedical Imaging (L Sugrue), University of California, San Francisco, CA; Center for Health Sciences (FC Baker), SRI International, Menlo Park, CA

**Keywords:** Adolescence, Cyberbullying, Media, Screens, Sleep

Digital media has become a central part of early adolescent life,^1^ bringing both benefits and risks. One such risk is cyberbullying, defined as aggressive or harmful behavior conducted through digital platforms intended to harass, threaten, or humiliate a victim. Cyberbullying in adolescents is associated with a lower likelihood of adequate sleep.^2^ Our original August 2023 publication entitled “Cyberbullying and Sleep Disturbance Among Early Adolescents in the U.S.” proposed bedtime screen use as a moderator between cyberbullying and sleep disturbances.^3^ In this progress report, we review recent findings on cyberbullying and sleep in early adolescence, with a focus on how disparities shape these experiences, and discuss their implications for adolescent health, family-based interventions, and equity-informed clinical practice.

## Disparities in Cyberbullying Exposure

Among 11 to 12-year-olds in the Adolescent Brain Cognitive Development (ABCD) Study, 9.6% report having experienced cyberbullying victimization.^1^ Sexual and gender minority adolescents experience higher rates of cyberbullying, with one study finding that transgender and gender-questioning early adolescents have more than twice the odds of cyberbullying victimization compared to their cisgender peers.^4^ Cyberbullying is associated with elevated risks of depression, anxiety, disordered eating, substance use, and suicidality.^1^ In addition to sexual/gender minority identity, adolescents who are racial/ethnic minorities or come from lower socioeconomic backgrounds are more likely to experience cybervictimization and online discrimination.^5^ Parental media behaviors, such as high screen use and lack of monitoring, are linked to problematic media use and higher cyberbullying exposure in their children.^6^

## Mechanisms for Disparities in Sleep Outcomes

There are strong correlations between sleep outcomes, race, and socioeconomic status.^7^ These include disparities in sleep quality and duration by socioeconomic status and disparities in quality and duration by race and ethnicity.^7^ Around 63% of early adolescents have an internet-connected device or TV in their bedroom, and behaviors such as social media use and keeping phone ringers on during the night are associated with lower sleep duration and higher sleep disturbances.^8^ Higher bedtime phone use is evident among sexual/gender minorities, racial/ethnic minorities, and low-income adolescents.^7,9^ Mechanisms contributing to poor sleep include blue light exposure, cognitive and emotional arousal from peer interactions, disruptions from notifications, and decreased association between the bedroom and sleep as a result of device presence in the sleep environment.^1,9^ Additional mechanisms involving phone use may explain cyberbullying and poor sleep outcomes.

## Linking Cyberbullying and Sleep

There is a suggested bidirectional and mutually reinforcing relationship between cyberbullying and sleep disturbance: cyberbullying can disrupt sleep, while poor sleep may increase vulnerability to peer conflict and emotional dysregulation.^1,9^ Adolescents exposed to cyberbullying may experience nighttime rumination, low self-esteem, shame, guilt, stress, and anxiety, all of which contribute to sleep disruption.^3^ These adolescents may also increase their screen use at night, further delaying or fragmenting sleep.^2,3^

Problematic social media use is central to both cyberbullying exposure and sleep disruption.^1,9^ Vulnerable populations, such as sexual/gender minority and racial/ethnic minority adolescents, may engage in more nighttime screen use seeking affirming or identity-specific spaces online.^1,9^ However, this also increases their risk of problematic screen use, cyberbullying victimization, and poor sleep outcomes.^1,9^

## Mental Health Implications

Problematic social media use, cyberbullying, and sleep problems are all linked to increased rates of mental and physical health conditions, including depression, anxiety, attention deficit hyperactivity disorder, obsessive-compulsive disorder, disordered eating, cardiometabolic risk, and substance use.^1^ These burdens are not equally distributed—adolescents with multiple marginalized identities face layered risks and compounding vulnerabilities.^1,9^

## Opportunities for Action and Intervention

Family-based strategies are promising for reducing risk across all domains. Interventions such as creating screen-free bedrooms, enforcing device curfews, and enhancing parental monitoring may help mitigate sleep disturbances, problematic screen use, and cyberbullying victimization.^1,9^ Recommendations for parents and physicians are summarized in Table.^1^ Interventions should be intersectional, while recognizing and addressing the interplay of identity, behavior, and structural context.^5^

## Future Work

Our original study established a link between cyberbullying involvement and sleep disturbances in early ado- lescence.^3^ Since then, additional research has validated and expanded these findings, suggesting that bedtime screen-use behavior connects both experiences, highlighting the impact of demographic disparities.^2,10^

Addressing these challenges will require a multi-level approach, integrating family education, clinical screening, and adolescent-centered behavioral interventions.^1,9^ Future research can leverage tools such as brain imaging to explore neurodevelopmental mechanisms and clarify the causal links between digital media use, cyberbullying, sleep disruption, and adolescent physical and mental health. Further research can examine whether sleep disturbances contribute to cyberbullying perpetration. In addition, more research is needed to assess whether school-enforced phone bans impact nighttime usage and sleep outcomes. As adolescents in the ABCD Study develop into mid- and late-adolescence, longitudinal data will enable researchers to examine shifts in cyberbullying exposure and perpetration over time.

Future work should additionally investigate protective and exacerbating factors among vulnerable adolescents, including those with multiple marginalized identities, to develop effective and equitable intervention strategies.

## Figures and Tables

**Table.** Recommendations for Parents and Physicians to Encourage Healthy Screen Use in Early Adolescence^1^

Recommendations for Parents	Recommendations for Physicians
Keep screens outside of the bedroom Limit parental screen use around children and model healthy screen use behaviors Restrict screen use during bedtime and mealtime Turn phones completely off overnight (versus silent/vibrate or keeping notifications on) Monitor adolescents’ screen use and set time limits Co-create an individualized Family Media Plan	Consider screening patients for sleep disorders Consider screening patients for signs of problematic or excessive social media use and cyberbullying Connect patients with culturally responsive mental health support

## What’s New

Recent scholarship has identified sociodemographic disparities in both cyberbullying and sleep outcomes in early adolescents. Cyberbullying is linked with poorer mental health, but parental rules and monitoring may be protective of adverse health effects.
